# Case report of heterotaxy syndrome with sinus node dysfunction and left ventricular hypertrabeculation: clinical and genetic insights

**DOI:** 10.47487/apcyccv.v6i3.496

**Published:** 2025-09-24

**Authors:** María Gabriela Matta, Prithviraj Dhonde, Edward Dababneh, Vaseekaran Gopalapillai, Clayton Sciberras, Kevin Ng, Nasser Mohamed Essack

**Affiliations:** 1 Department of Cardiology, Division of Specialist Medical Services, Gold Coast Hospital and Health Services, Southport, QLD 4215, Australia Department of Cardiology Division of Specialist Medical Services Gold Coast Hospital and Health Services Southport Australia

**Keywords:** Heterotaxy Syndrome, Sick Sinus Syndrome, Ventricular Dysfunction, Genetic Diseases, Síndrome de Heterotaxia, Síndrome del Seno Enfermo, Disfunción Ventricular, Enfermedades Genéticas

## Abstract

We present the case of a 41-year-old woman with left atrial isomerism, severe sinus node dysfunction, and left ventricular hypertrabeculation, who required implantation of a dual-chamber implantable cardioverter-defibrillator with left bundle branch area pacing. Her family history revealed multiple cases of heterotaxy and conduction disorders. Genetic testing identified a heterozygous interstitial duplication on chromosome 17q23.2 involving the MED13 gene, whose clinical significance has not yet been determined.

## RESUMEN

Presentamos el caso de una mujer de 41 años con isomerismo auricular izquierdo, disfunción severa del nodo sinusal e hipertrabeculación del ventrículo izquierdo, quien requirió la implantación de un desfibrilador automático implantable bicameral con estimulación en la región de la rama izquierda del haz de His. Su historia familiar reveló múltiples casos de heterotaxia y trastornos de la conducción. El estudio genético identificó una duplicación intersticial heterocigota en el cromosoma 17q23.2 que incluye el gen *MED13*, cuya implicancia clínica aún no ha sido determinada.

## Introduction

Heterotaxy syndrome, or situs ambiguus, is a rare congenital disorder affecting the lateralization of thoracoabdominal organs, with an estimated prevalence of 1 in 10,000 live births. It is characterized by a spectrum of cardiac and extracardiac anomalies, including intestinal malrotation, polysplenia or asplenia, and venous anomalies, which reflect the complex interplay of genetic and embryological factors during organ development. Cardiac manifestations commonly include septal defects, atrioventricular canal defects, and atrial isomerism, with inheritance patterns that range from sporadic to various forms of genetic transmission. ^1^^-^^3^

Beyond structural anomalies, heterotaxy is associated with sinoatrial dysfunction and left ventricular hypertrabeculation (LVHT, previously referred to as left ventricular non-compaction), a myocardial disorder that predisposes individuals to arrhythmias, systolic dysfunction, and thromboembolism. Although mutations in several genes have been implicated in both heterotaxy and LVHT, the genetic basis underlying their co-occurrence remains incompletely understood^4^^-^^8^.This report presents a case of heterotaxy syndrome with sinus nodal dysfunction and LVHT, adhering to the CARE guidelines for case reporting.

## Case report

A 41-year-old physically active woman who regularly attended the gym and had no major cardiovascular risk factors presented to the emergency department with lightheadedness, palpitations, and shortness of breath. Her medical history included a diagnosis of left atrial isomerism, confirmed by cardiac magnetic resonance imaging (MRI) and thoracoabdominal computed tomography (CT), which demonstrated polysplenia, intestinal malrotation, and azygos continuation of the inferior vena cava (IVC). She also had longstanding sinoatrial disease with bradycardia, which had been asymptomatic and managed conservatively. Her family history was notable for a mother diagnosed with atrial fibrillation at 40 years old and pulmonary stenosis. She also had two daughters, aged 8 and 10, both with malrotation syndrome, polysplenia, and interrupted IVC. One of her daughters required a pacemaker at age 10 due to conduction abnormalities.

Approximately six years prior to presentation, the patient underwent a comprehensive evaluation for persistent bradycardia and a family history of arrhythmias. An exercise stress test performed at that time demonstrated a workload of 12 METs, with a maximum heart rate of 145 bpm (70% of the predicted value). The exercise electrocardiogram (ECG) revealed a junctional rhythm with high-grade atrioventricular (AV) dissociation, along with frequent premature ventricular contractions (PVCs) and ventricular couplets during exertion.

Genetic testing ruled out pathogenic mutations in genes associated with sodium channelopathies and other inherited arrhythmia syndromes. Additional investigations, including a cardiac gene panel and laterality gene analysis, were negative for clinically significant variants. A single nucleotide polymorphism (SNP) array identified a variant of unknown clinical significance (VUS). Transthoracic echocardiography at that time demonstrated a left ventricle of normal size and function, with apical trabeculations and no evidence of valvular disease. Cardiac MRI also showed left ventricular hypertrabeculation; however, the findings did not fulfill diagnostic criteria for LVHT. Further genomic analysis using the Illumina Infinium CytoSNP 850K Array revealed a heterozygous interstitial duplication at chromosome 17q23.2 involving a portion of the MED13 gene.

**Figure 1:**
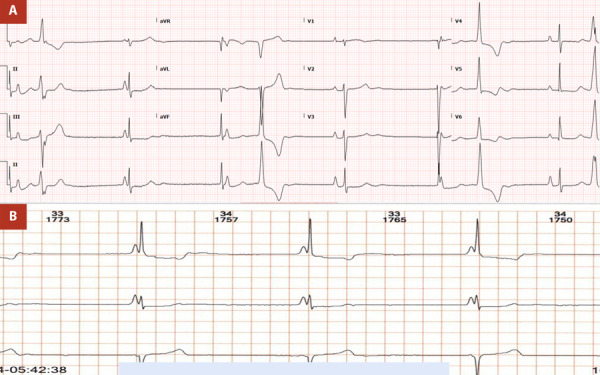
A. ECG showing isorhythmic AV dissociation and bifocal PVCs. B. Holter monitoring showing isorhythmic AV dissociation, with both sinus node and junctional escapes at similar rates.

At the time of the current presentation, the ECG revealed junctional bradycardia with functional AV dissociation consistent with isorhythmic AV dissociation, indicative of severe sinus node dysfunction. Other findings included bifocal PVCs, a QRS duration of 60 msec, and right-axis deviation without evidence of right bundle branch block (RBBB) (Figure 1A). A Holter monitor conducted earlier in the year showed similar findings, including isorhythmic AV dissociation (Figure 1B). Continuous telemetry during her hospital stays confirmed persistent isorhythmic AV dissociation, severe junctional bradycardia, bifocal PVCs, and short runs of ventricular bigeminy, without episodes of non-sustained ventricular tachycardia or pauses exceeding 2.5 seconds. Laboratory findings were unremarkable.

**Figure 2:**
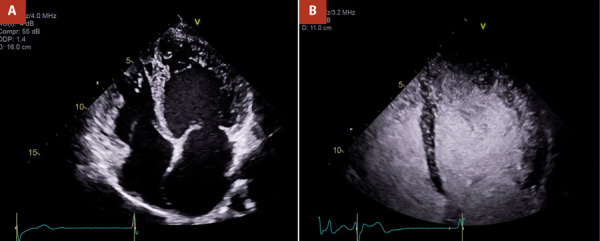
Echocardiogram in apical four-chamber view without **(A)** and with contrast **(B)** showing left ventricular hypertrabeculation, dilatation, and mildly impaired left ventricular systolic function.

A contrast-enhanced echocardiogram demonstrated a moderately dilated left ventricle with prominent trabeculations, now meeting criteria for LVHT. The ratio of noncompacted to compacted myocardium exceeded 2.3 at end-systole, primarily involving the inferolateral walls and apex per Jenni criteria, with additional right ventricular involvement. Left ventricular ejection fraction (EF) was mildly reduced (~45%), and the right ventricle was dilated but maintained normal systolic function (Figure 2).

**Figure 3:**
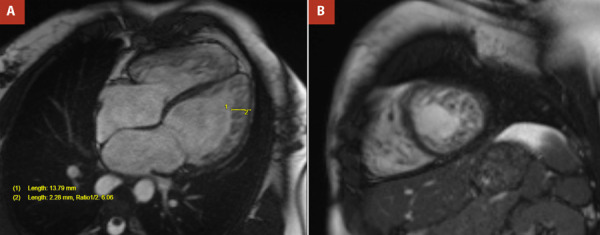
Cardiac MRI. Cine images in four chambers **(A)** and short-axis mid-ventricular **(B)** showing hypertrabeculation in the right and left ventricles.

**Figure 4:**
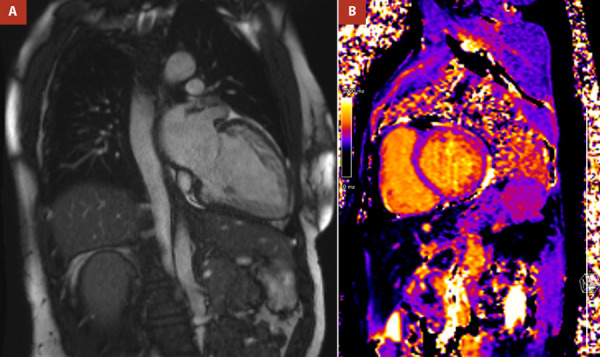
Cardiac MRI. Cine images in two chambers showing hypertrabeculation **(A)** and native T1 mapping **(B)** showing normal values.

Cardiac MRI confirmed the diagnosis of LVHT, revealing hypertrabeculation in the anterolateral, inferolateral, and inferior walls, with a noncompacted-to-compacted ratio ≥6:1 at end-diastole in multiple segments. More than 25% of the left ventricular myocardial mass was classified as noncompacted. No gadolinium enhancement was observed, ruling out myocardial fibrosis (Figures 3A, 3B, and 4A). Native T1 mapping values were within normal limits (Figure 4B). Left ventricular function was mildly reduced, with an EF of 45%. No valvular abnormalities were identified.

After a shared decision-making process and following a multidisciplinary Heart Team discussion, the patient underwent successful implantation of a dual-chamber implantable cardioverter-defibrillator (ICD) configured for left bundle branch area pacing. This decision was based on two key considerations: first, the presence of chronotropic incompetence demonstrated on stress echocardiography, which confirmed the need for reliable pacing support; and second, concern for increased arrhythmic risk given her underlying structural heart disease and electrocardiographic abnormalities, prompting prophylactic ICD implantation.

Despite the technical challenge of a thin interventricular septum (6 mm), deep septal left ventricular pacing was successfully achieved, minimizing the risk of pacing-induced cardiomyopathy associated with right ventricular pacing. Defibrillator and right atrial leads were also appropriately positioned. Device testing confirmed 1:1 AV conduction up to 130 beats per minute. Post-procedural ECG demonstrated satisfactory atrial pacing (Figure 1S- supplementary material).

The patient was discharged in stable condition and scheduled for follow-up in the electrophysiology clinic.

## Discussion

Left atrial isomerism, as seen in this patient, is distinct from its right-sided counterpart, which is frequently associated with asplenia and a higher risk of complete atrioventricular block. In contrast, left atrial isomerism is more commonly linked to sinoatrial dysfunction and polysplenia. The abnormal atrial arrangement disrupts normal sinus node function. While both subtypes can feature twin atrioventricular nodes and shifting P waves, the absence of significant atrioventricular conduction disease in left atrial isomerism differentiates it from right atrial isomerism. ^1^^-^^5^

Heterotaxy and LVHT have also been linked. A retrospective review reported the presence of LVHT in 7.5% of patients with heterotaxy. ^6^ Although several genes associated with heterotaxy have been identified-such as *MMP21, C1orf127, CCDC39, CIROP, DNAAF3, DNAH5, DNAH9*, *MNS1*, *ZIC3, CFC1,* and *GDF1*-the genetic basis of heterotaxy remains elusive in most cases. ^7^^-^^10^ A potential linkage on chromosome 6p has been suggested, but a specific gene directly explaining the co-occurrence of LVHT and heterotaxy has not been identified. ^11^ Similarly, while sinus node dysfunction can be associated with mutations in genes involved in cardiac conduction (e.g., *HCN4*), its relationship with LVHT remains unclear. ^5^^,^^12^^-^^14^ A summary of known genetic mutations associated with LVHT and sinus node dysfunction is provided in Supplementary Tables 1S and 2S, respectively. Notably, the genes implicated in these three conditions-heterotaxy, LVHT, and sinus node dysfunction-are distinct, further underscoring the complexity of their genetic interplay.

**Figure 5:**
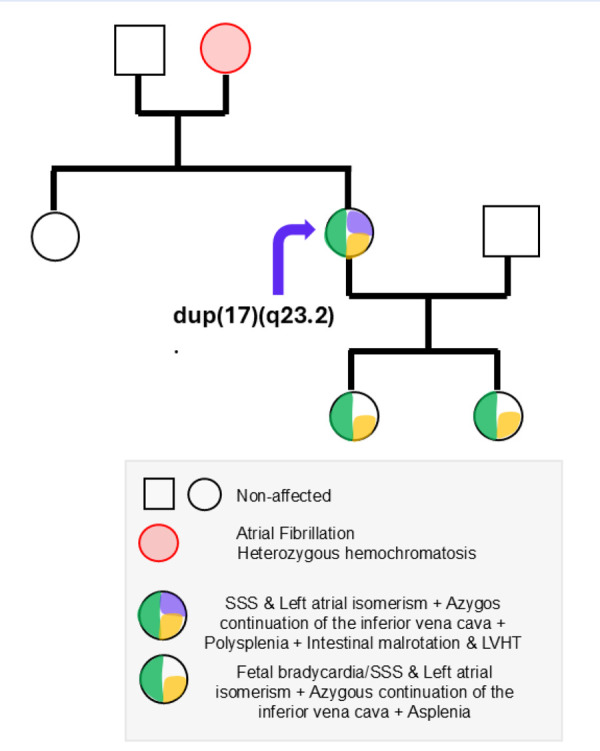
Pedigree showing the familial inheritance of congenital cardiac and visceral anomalies associated with a 17q23.2 duplication. The proband (purple arrow) carries a duplication at 17q23.2 and presents with sinus node dysfunction (SSS), left atrial isomerism, azygos continuation of the inferior vena cava, polysplenia, intestinal malrotation, and left ventricular hypertrabeculation (LVHT). Her child exhibits fetal bradycardia, SSS, left atrial isomerism, azygos continuation of the inferior vena cava, and asplenia. The proband’s mother has a history of atrial fibrillation and is heterozygous for hemochromatosis. Other family members are unaffected. Circles represent females; squares represent males. Filled segments within symbols indicate specific clinical features as detailed in the legend.

In our patient, genetic testing for sodium channelopathies and laterality, including SCN5A mutations, was negative. Genetic testing for the patient’s two daughters was also negative for channelopathies. Microarray analysis was not conducted on the mother, and both daughters have normal genetic test results. The only genomic variation identified in the patient was a heterozygous interstitial duplication at chromosome 17q23.2. This duplication encompasses part of the MED13 gene, a component of the Mediator complex, which plays a crucial role in regulating gene transcription during development. Figure 5 shows the family pedigree.

Although *MED13* has not been directly linked to heterotaxy, sinus node dysfunction, or LVHT in current literature or genetic databases ^15^^,^^16^, its involvement in developmental pathways suggests a possible, albeit unproven, contribution to the patient’s phenotype. Notably, a de novo missense variant in *MED13* was recently reported in an infant with congenital heart anomalies and multiple developmental abnormalities, raising the possibility of a broader role for this gene in cardiac morphogenesis. ^15^

Management of sinus node dysfunction in the setting of heterotaxy and LVHT must be personalized, balancing the severity of conduction abnormalities and arrhythmia risk. ^17^ Although current ESC guidelines do not provide specific recommendations for ICD implantation in patients with LVHT and a left ventricular ejection fraction greater than 35%, the evidence regarding ICD use in LVHT patients remains limited. ^17^^-^^19^ In this case, the combination of chronotropic incompetence, arrhythmic risk, and the patient’s genetic profile, the decision was made to proceed with the ICD implantation as a preventive measure against sudden cardiac arrest.

Left bundle branch pacing was selected to reduce the risk of pacing-induced cardiomyopathy, a known complication of traditional right ventricular pacing, particularly in patients with left ventricular dysfunction. ^20^ This approach aims to provide effective bradycardia support while mitigating potential long-term adverse effects on ventricular function, thereby improving the patient’s overall prognosis.

In conclusion, this case exemplifies the complexity of managing patients with heterotaxy syndrome, particularly when coexisting with sinus node dysfunction and LVHT. It underscores the importance of individualized care that incorporates detailed anatomical, electrophysiological, and genetic evaluation. Notably, this is the first report-based on current literature-to identify a duplication involving the *MED13* gene in a patient presenting with this triad of conditions. While the pathogenicity of this variant remains uncertain, its involvement in developmental transcriptional regulation raises the possibility of a novel genetic contribution to the heterotaxy-LVHT-sinus node dysfunction spectrum. This finding highlights the need for further investigation into the role of *MED13* in cardiac laterality, morphogenesis, and conduction system development.
